# Comprehensive chemical profiling of two *Dendrobium* species and identification of anti-hepatoma active constituents from *Dendrobium chrysotoxum* by network pharmacology

**DOI:** 10.1186/s12906-023-04048-y

**Published:** 2023-07-01

**Authors:** Xia Jie, Yin Feng, Fang Jiahao, Lou Ganggui, Yu Jiani, Xu Zhongyu, Yuan Yuan, Zhang Tinggang, Zhang Xiaodan, Liang Zongsuo

**Affiliations:** 1 College of Life Sciences and Medicine, Key Laboratory of Plant Secondary Metabolism and Regulation of Zhejiang Province, Zhejiang Sci-Tech University, Hangzhou, China; 2 Zhejiang Sci-Tech University Shaoxing Academy of Biomedicine Co., Ltd, Shaoxing, China; 3 State Key Lab Breeding Base Dao-Di Herbs, National Resource Center Chinese Materia Medica, Beijing, China Academy of Chinese Medical Sciences, Beijing, China; 4 Yunnan YunHu Biotechnology Co., Ltd, Wenshan, China

**Keywords:** *D. nobile*, *D. chrysotoxum*, Metabolomics, Network pharmacology, Hepatoma, Biomarkers

## Abstract

**Background:**

*Dendrobium nobile* and *Dendrobium chrysotoxum* are important species of the genus *Dendrobium* and have great economic and medicinal value. However, the medicinal properties of these two plants remain poorly understood. This study aimed to investigate the medical properties of *D. nobile* and *D. chrysotoxum* by conducting a comprehensive chemical profiling of the two plants. Additionally, active compounds and predictive targets for anti-hepatoma activity in *D. chrysotoxum* extracts were identified using Network Pharmacology.

**Results:**

Chemical profiling showed that altogether 65 phytochemicals were identified from *D. nobile* and *D. chrysotoxum*, with major classes as alkaloids, terpenoids, flavonoids, bibenzyls and phenanthrenes. About 18 compounds were identified as the important differential metabolites in *D. nobile* and *D. chrysotoxum*. Furtherly, CCK-8 results showed that the extracts of stems and leaves of *D. nobile* and *D. chrysotoxum* could inhibit the growth of Huh-7 cells, and the anti-hepatoma activity of extracts were dose-dependent. Among the extracts, the extract of *D. chrysotoxum* showed significant anti-hepatoma activity. In order to find the potential mechanism of anti-hepatoma activity of *D. chrysotoxum*, five key compounds and nine key targets were obtained through constructing and analyzing the compound-target-pathway network. The five key compounds were chrysotobibenzyl, chrysotoxin, moscatilin, gigantol and chrysotoxene. Nine key targets, including GAPDH, EGFR, ESR1, HRAS, SRC, CCND1, HIF1A, ERBB2 and MTOR, could be considered as the core targets of the anti-hepatoma activity of *D. chrysotoxum*.

**Conclusions:**

In this study, the chemical composition difference and anti-hepatoma activity of stems and leaves of *D. nobile* and *D. chrysotoxum* were compared, and the potential anti-hepatoma mechanism of *D. chrysotoxum* was revealed in a multi-target and multi-pathway manner.

**Supplementary Information:**

The online version contains supplementary material available at 10.1186/s12906-023-04048-y.

## Introduction

Hepatoma is the major cause of cancer associated mortality. In fact, since cancer treatment is often accompanied by side effects, there is a growing demand for innocuous and more effective anticancer drugs [1]. Phytochemicals isolated from medicinal plants have been largely neglected in this context, although their pharmacological activities have been well investigated in the past, and they may have considerable medicinal potential [2]. In particular, medicinal plants could be and more effective in the treatment of various diseases, including cancer [3]. Various biomolecules presented in the plant extract such as alkaloids, terpenoids, polyphenols, polysaccharides, flavonoids, tannins, saponins, phenolics, amino acids, and proteins are natural sources of therapeutic drugs [4–8].

**Fig. 1:**
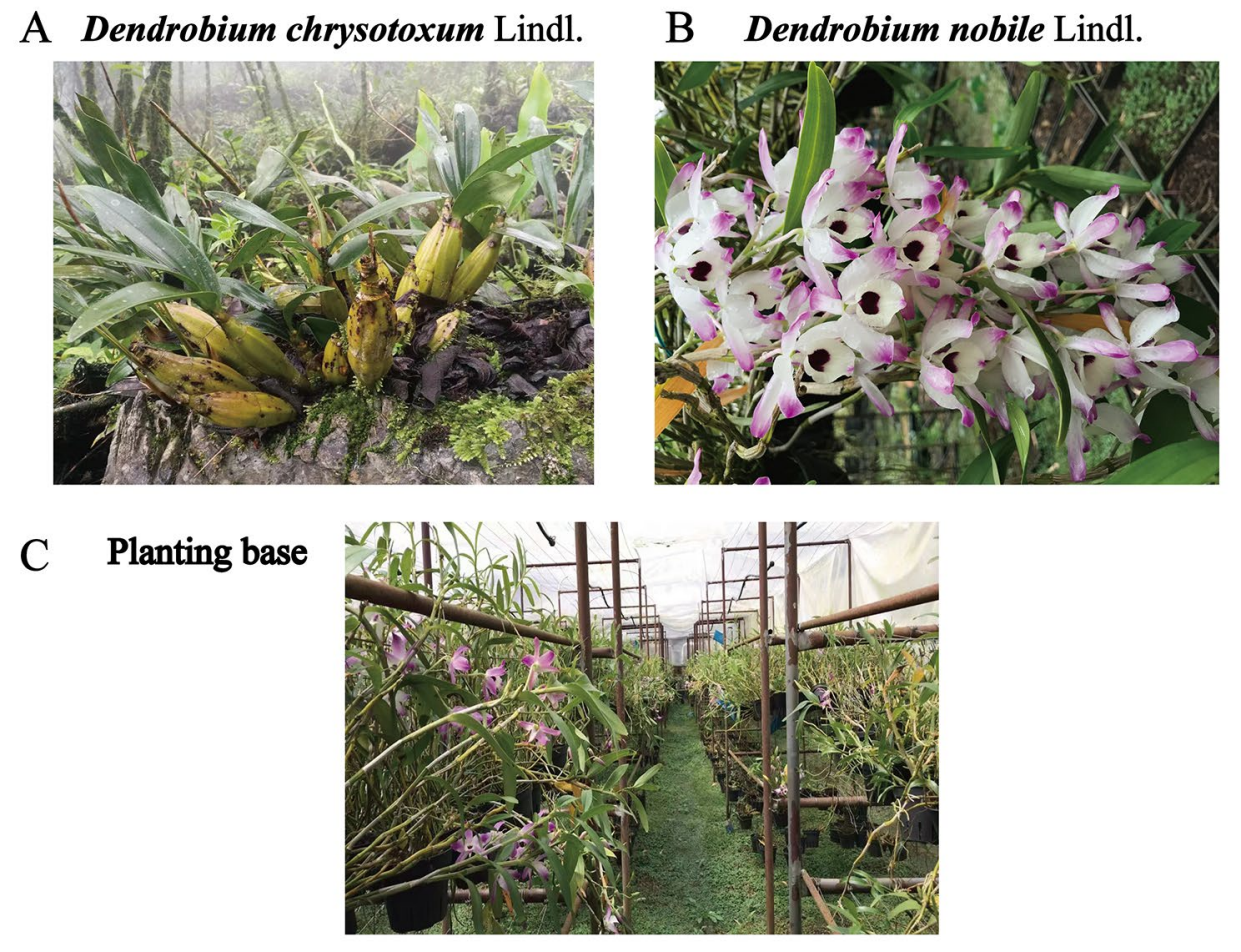
Plants and metabolites. (**A**, **B**, **C**) *Dendrobium* species planting base in Wenshan, Yunnan

With approximately 1,400 native species, *Dendrobium* is one of the largest families of orchids and is widely distributed around the world [9]. Recent pharmacological studies have shown that *Dendrobium* has a wealth of medicinal effects, such as hepatoprotective, anti-inflammatory, anti-angiogenic and anti-oxidative properties [9, 10]. The stems of *D. nobile* and *D. chrysotoxum* have great economic and medicinal value and are listed in the Chinese Pharmacopoeia of 2020 (Fig. 1A, B and C) [11]. The chemical constituents of *D. nobile* and *D. chrysotoxum* are mainly alkaloids, terpenoids, polyphenols, flavonoids, phenanthrenes, bibenzyls and amino acids [12–14]. Recent pharmacological studies have shown that components of the stems of *Dendrobium*, possess a broad range of activities, encompassing hepatoprotective, anti-proliferative activity toward cancer cells, immunostimulating, anti-diabetic activity, cataractogenesis-inhibiting activity, neuroprotective activity, anti-inflammatory activity, anti-platelet aggregation activity and hemagglutininating activities and also exert beneficial actions on colonic health and alleviate symptoms of hyperthyroidism [15–17]. Because only the stems of *D. nobile* and *D. chrysotoxum* are permitted for use according to the Chinese Pharmacopoeia, their leaves are largely discarded [18]. However, the leaves of these *Dendrobium* plants possess chemical constituents similar to those found in the stems [19–21].

Plant extracts act on certain molecular targets due to the synergistic effects of their chemical compounds and the fact that they could interact with many targets simultaneously [3, 22]. In recent years, network pharmacological analysis has been effectively applied to predict the relationship between protein targets, active compounds and related diseases. This approach attempts to explore disease-related, multiple active compounds and targets, thus allowing to characterize the multi-target mechanism of bioactive compounds [23, 24]. This analysis can provide insights into the link between Traditional Chinese Medicine (TCM) bioactive compounds and diseases, possibly revealing the pharmacological properties of multiple biomolecules found in medicinal plants [25].

Screening and identification of active components from TCM is rather challenging due to the diversity and complexity of chemical components. Herein, comprehensive metabolite profiling of the *D. nobile* and *D. chrysotoxum* were conducted by LC-MS using an untargeted metabolomics approach. The anti-hepatoma activities of the extracts from the stems and leaves of *D. Nobile* and *D. chrysotoxum* were compared by CCK-8 assay. A network pharmacology approach was applied to characterize the possible mechanism of action of *D. chrysotoxum* compounds on the human hepatoma Huh-7 cell line. Active compounds and potential targets of *D. chrysotoxum*, as well as related genes of hepatoma were obtained from the public databases, while the potential targets and signalling pathways were determined by protein-protein interaction (PPI), gene ontology (GO) and pathway enrichment analyses. Ultimately, the compound-target and target-pathway networks were constructed. This study compared the chemical composition and anti-hepatoma activity of *D. nobile* and *D. chrysotoxum* extracts, and revealed the potential anti-hepatoma action mechanism of *D. chrysotoxum* using a multi-target and multi-pathway approach.

## Materials and methods

### Reagents and materials

Methanol (MeOH), acetonitrile and ethanol were obtained from Thermo Fisher Scientific (Waltham, MA, USA). 5-Fluorouracil was bought from Macklin Biochemica Co., Ltd.(Shanghai, China). CCK-8 kit was bought from Beyotime Biotechnology(Shanghai, China); Phosphate Buffered Saline (PBS) was bought from Biological Industries (The State of Israel); DMEM medium; Fetal bovine serum (FBS); Antibiotics; Trypsin were bought from Thermo Fisher Scientific (Massachusetts, US).

*D. nobile* and *D. chrysotoxum* were harvested from Wenshan, Yunnan Province, China. The experiment was conducted using at 3-year-old wild-cultured *D. nobile* and *D. chrysotoxum*, which were collected in February.

Human hepatocellular carcinoma (Huh7 cell line) was purchased from Hangzhou Qiannuo Biotechnology Co., Ltd (Yuanlong Commercial Building, Baiyang Street, Qiantang District, Hangzhou, China). Normal human hepatocytes (Lo2 cells line) was purchased from Shanghai Cell Bank, Chinese Academy of Sciences (No. 320 Yueyang Road, Xuhui District, Shanghai, China). ( Note:We used only Huh-7 and Lo2 (normal human hepatocytes) cell lines in our experiments, and no other human samples were used.)

### Extraction procedure

The stems and leaves of the *D. nobile* and *D. chrysotoxum* were cut into small segments completely dried at 50–60 ℃. Then crushed by a pulverizer and put into a sealed bag for storage. 10 g of crushed stems and leaves were extracted with 500 mL of 70% methanol aqueous solution with ultrasonic for 45 min respectively, the filtrate was collected, concentrated by a rotary evaporator, and freeze-dried [26].

### Liquid chromatography tandem mass spectrometry (LC–MS/MS) analysis for metabolite identification

#### Sample preparation for metabolomics

The tissue samples were dried at 55℃ and ground into a fine powder. Approximately 0.01 g of root was homogenized with 10 mL of MeOH/H_2_O (70:30, v/v), centrifuged for 5 min and subjected to ultrasonic treatment for 20 min. The sample was then centrifuged at 10,000 rpm for 10 min, and the resulting supernatant solution was passed through a 0.22-µm pore-size membrane. The filtrate was transferred to sample vials for LC-MS/MS analysis [26].

#### UPLC-Q-TOF-MS/MS analysis method

Chromatographic separation was achieved using an Waters ACQUITY UPLC HSS T3 column (1.8 μm, 2.1 mm × 100.0 mm; Waters Corporation, Milford, MA, USA). The column temperature and flow rate were set at 40℃ and 0.4 mL·min^− 1^, respectively. The injection volume and detection wavelength of the chromatographic column were set at 1 µL and 254 nm, respectively. The mobile phase was composed of water (A) and acetonitrile (B), with a gradient elution program of 4% B (0–1 min), 4–50% B (1–7 min), 50–70% B (7–8 min), 70–95% B (8–12 min), 95% B (12–15 min), 95 − 4% B (15–16 min) and 4% B (16–18 min).

Mass spectrometric detection was performed in electrospray mode using a Xevo G2-XS Q-Tof mass spectrometer detector (Waters Corporation, Milford, MA, USA). Argon was used as the desolvation and collision gas. The full-scan data range was 50 − 1,200 Da, the source temperature was 100 °C, the desolvation temperature was 400 °C and the scanning frequency was 1.000 s. The locking spray standard was 400 mg·mL^− 1^ with a collision energy of 6.000 V for the low collision energy scan and 30 to 70 V for the high collision energy scan of the mass spectrometer, and the UPLC system was controlled by MassLynx4.1 software (Waters Corporation, Milford, MA, USA).

### CCK-8 assay of plant extracts and cytotoxicity tests

Human hepatoma Huh-7 cells were maintained routinely in DMEM supplemented with 10% FBS and 1% antibiotics in a humidified atmosphere of 5% CO_2_/50% air [27]. The anti-hepatoma activity of extracts from the stems and leaves of the *D. nobile* and *D. chrysotoxum* were determined by CCK-8 assay. 100 µL cell suspension were cultured in a 96-well plate for 24 h, then the medium was removed, and the cells were incubated with different concentrations of the extracts, the extracts were dissolved in DMEM medium containing 10% FBS and 1% antibiotics for 48 h. 10 µL CCK-8 solution was added to each well for 2 h, the absorbance was measured at 450 nm using a microplate reader. The absorbance of blank group without cells was Abs blank. 5-Fluorouracil was used as a positive control [28]. The cell inhibition rate of extract solution was calculated according to the following formula.

$${\rm{Inhibition}}\,\,{\rm{rate}}\,\left( {\rm{\% }} \right){\rm{ = }}\,{\rm{1 - }}\frac{{{\rm{Ab}}{{\rm{s}}_{{\rm{sample}}}}{\rm{ - Ab}}{{\rm{s}}_{{\rm{blank}}}}}}{{{\rm{Ab}}{{\rm{s}}_{{\rm{control}}}}{\rm{ - Ab}}{{\rm{s}}_{{\rm{blank}}}}}}{\rm{ \times 100}}$$

To evaluate the effect of *D. nobile* and *D. chrysotoxum* on the viability of Lo2 cells (normal human hepatocytes), cytotoxicity assays were performed using the Cell Counting Kit-8. We used the method of Xia et al. as a reference for experimental design, briefly, cells in the logarithmic growth phase were subjected to the cell passage cultivation and finally 100 µL of cell suspension (cell density of 1 × 10 ^5^ /mL) was added to each well of the 96-well plate. The blank control was cell-free cell culture medium. The final concentrations of the experimental group were 0, 50, 100, 150, 200, 250, 500 µg/mL, and 6 replicate wells were added for each concentration, and each group was repeated 3 times. For the control group, only 100 µL of cell culture medium was added. After incubation for 24 h, 10 µL of CCK-8 was added to each well and incubated for 2 h. The absorbance (OD) was measured at 450 nm on an enzyme marker [29].

$${\rm{Survival}}\,\,{\rm{rate}}\,\left( {\rm{\% }} \right){\rm{ = }}\,{\rm{1 - }}\frac{{{\rm{O}}{{\rm{D}}_{{\rm{sample}}}}{\rm{ - O}}{{\rm{D}}_{{\rm{blank}}}}}}{{{\rm{O}}{{\rm{D}}_{{\rm{control}}}}{\rm{ - O}}{{\rm{D}}_{{\rm{blank}}}}}}{\rm{ \times 100}}$$

### Network pharmacology analysis

#### Database construction and prediction of potential targets

The SIMILES and InChIKey of the chemical components of aerial part of *D. chrysotoxum* were collected from the PubChem database. The GI absorption and bioavailability score of the compounds were calculated by SwissADME, and the compounds with GI absorption were high and bioavailability score > 0.3 were screened. The Swiss Target Prediction database was used to obtain the target corresponding of the components [30]. Use “Liver cancer” as the key words, the liver cancer related genes were screened out by DisGeNET [31], GeneCards [32], OMIM [33]. The obtained targets of the constituents and the disease targets of liver cancer were unified as UniProt ID through the UniProt database [34].

#### Network construction

Cytoscape software was used to construct the “drug components-target” network to explain the interactions between the core chemical components of *D. chrysotoxum* and potential targets in liver cancer therapy. In order to better analyze the protein-protein interaction, the PPI network of potential targets was constructed by using the STRING database using the common target of the liver cancer related genes and target corresponding of the components, the species was defined as Homo sapiens [35]. The “drug components-target” network and PPI network was imported into Cytoscape software for topological attribute analysis, and the key nodes and degree values in the network map were analyzed to obtain the core components and targets of *D. chrysotoxum*.

#### Biological function and pathway analysis

In order to illustrate the role of the core target in the gene function and signal pathway of the active ingredient in the treatment of liver cancer. David database was used to perform gene ontology (GO) function and Kyoto Encyclopedia of Genes and Genomes (KEGG) pathway enrichment analysis to explore the mechanism of anti-hepatoma activity of *D. chrysotoxum* [36–39].

### Data analysis

We used the method of Xia et al. as a reference for data analysis, mass spectrometry data were collected using MassLynx V4.2 software (Waters Corporation, Milford, MA, USA) for automatic peak identification, peak matching, peak alignment, peak extraction, peak integration and normalization. The metabolites and their possible cleavage modes were identified using secondary mass spectrometry data, Unifi (Waters Corporation, Milford, MA, USA), online databases (SciFinder, Chemspider and PubMed) and data from previous studies. Unsupervised principal component analysis (PCA) and supervised orthogonal partial least squares discriminant analysis (OPLS-DA) were performed using SIMCA-P 14.1 (Umetrics Corporation, Umea, Sweden) software [29].

## Results

### Overview of the metabolomes of different tissues

**Fig. 2:**
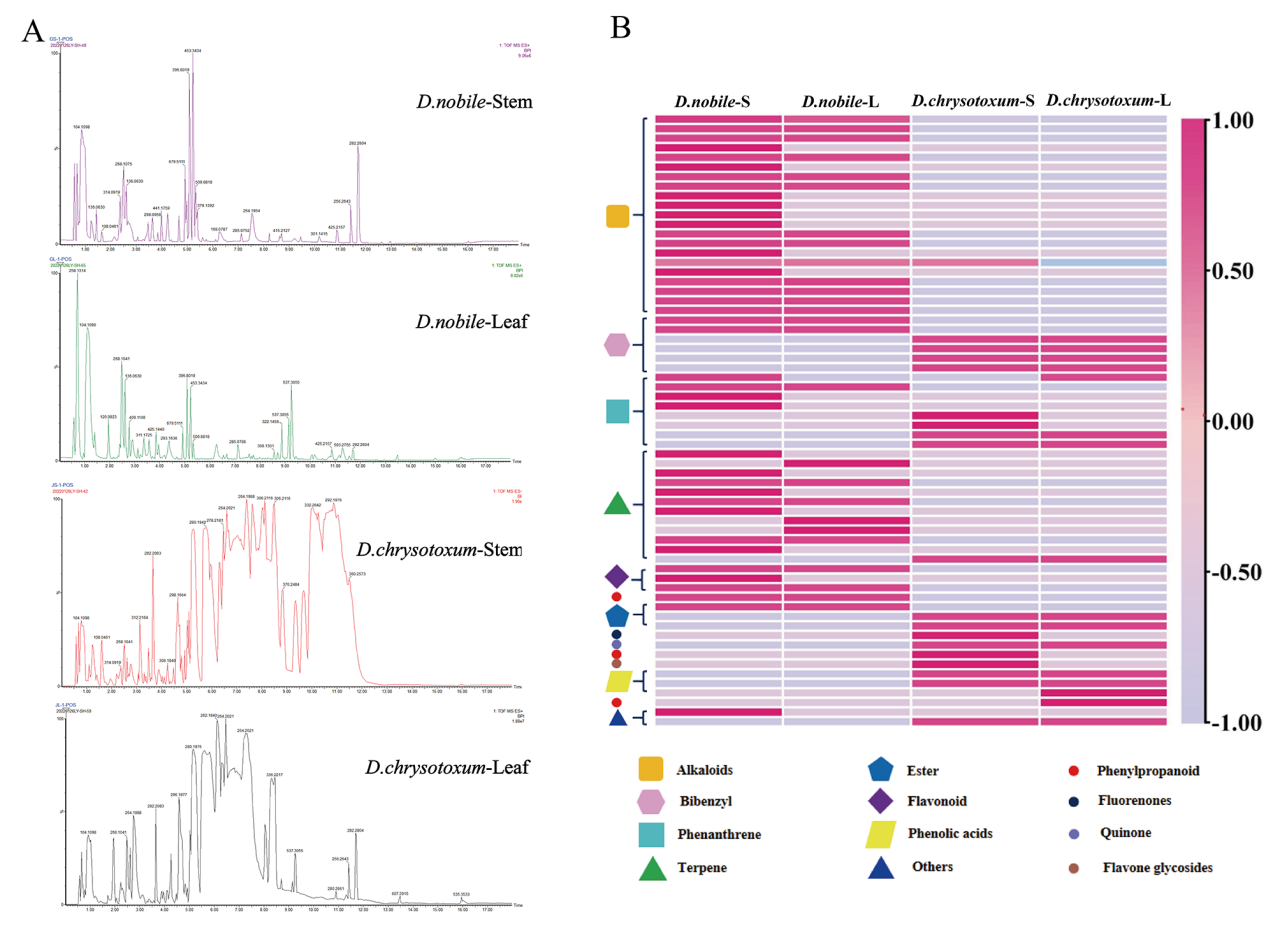
Differentially accumulated metabolites among the four tissues of two Dendrobium species. (**A**)The base peak ion chromatogram (BPI) of different samples; (**B**) A heatmap of the abundance of metabolites in the four tissues of two Dendrobium species

Comprehensive metabolite profiling of the *D. nobile* and *D. chrysotoxum* was conducted by LC-MS using an untargeted metabolomics approach. By observing the trend of ion peaks in BPI and the clustering of metabolite species between samples, it was indicated that there was a high similarity in the accumulation of metabolites between different tissues of the same *Dendrobium* species (Fig. 2A and B). In order to analyze the differences of accumulated metabolites between different samples, the identified metabolite types were assigned to different samples (Fig. 2B). In *D. nobile*, the major metabolite types were alkaloids (21 metabolites), terpenoids (8 metabolites), flavonoids (3 metabolites) and phenanthrenes (3 metabolites); in *D. chrysotoxum*, the largest number of metabolite types belonged to bibenzyls (4 metabolites), phenanthrenes (3 metabolites) and phenolic acids (2 metabolites) (Figure S1). Notably, As for the metabolites specifically enriched in *D. nobile*, alkaloids were abundant in stems but also were observed in leaves (Fig. 2B). In both plants, the predominant differential metabolites were alkaloids and bibenzyls.

### Metabolomic multivariate statistical analysis of *D. nobile* and *D. chrysotoxum*

#### Metabolic profiling of *D. nobile* and *D. chrysotoxum*

**Fig. 3:**
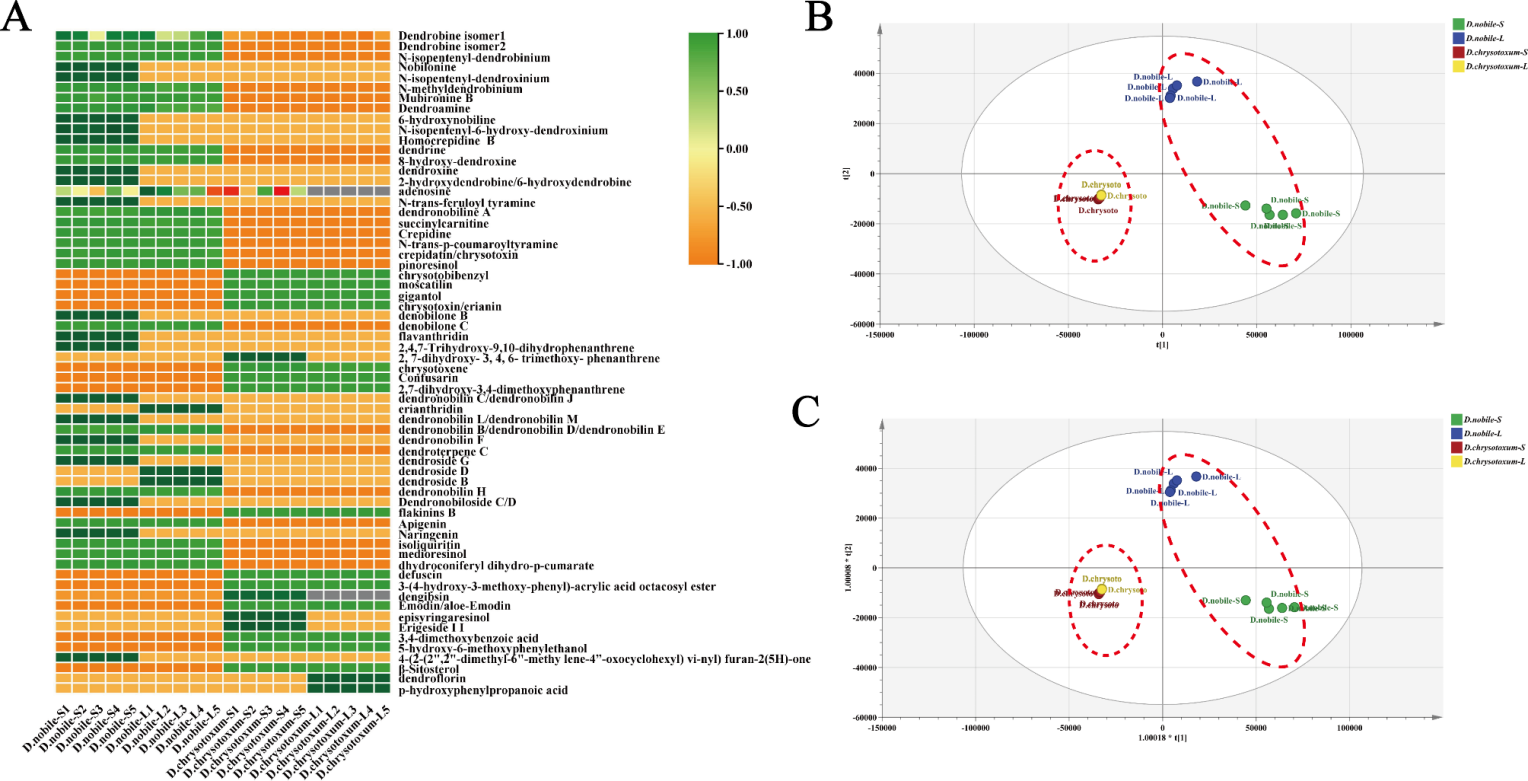
The results of non-targeted metabolomics and chemometric analysis of *D. nobile* and *D. chrysotoxum.* (**A**) Heat map of metabolomics and chemometrics clustering of *D. nobile* and *D. chrysotoxum*; (**B**) Principal component analysis of *D. nobile* and *D. chrysotoxum*; (**C**) OPLS-DA analysis of *D. nobile* and *D. chrysotoxum* (*D. nobile-*S: *D. nobile* stem;*D. nobile-*L: *D. nobile* leaf; *D. chrysotoxum-*S: *D. chrysotoxum* stem; *D. chrysotoxum-*L: *D. chrysotoxum* leaf; No.1–5: Number of repetitions)

**Fig. 4:**
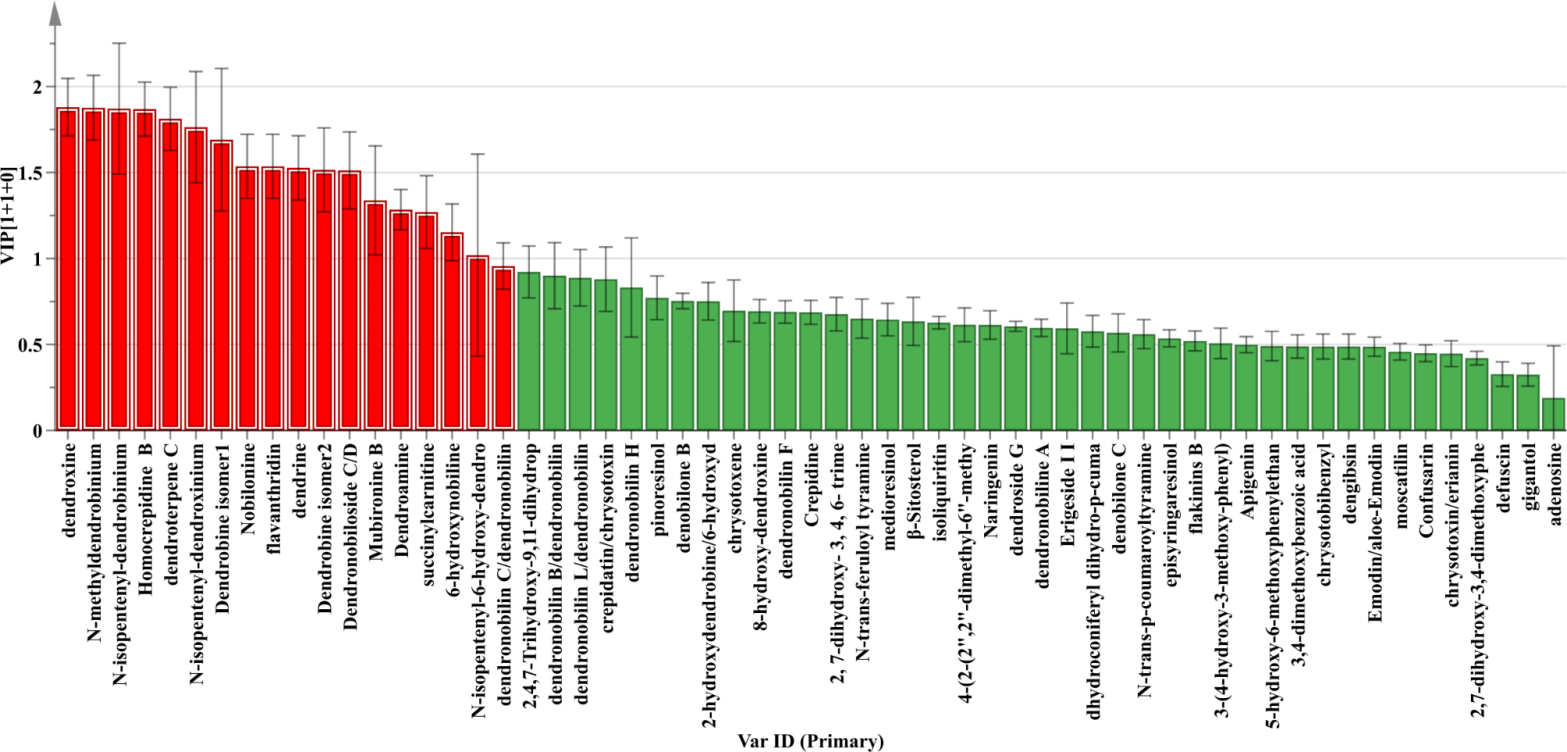
The values of VIP of the *D. nobile* and *D. chrysotoxum*

Using our in-house and public databases, we tentatively identified 65 metabolites of negative and positive ion modes, and thus gave a comprehensive metabolome for the stems and leaves of *D. nobile* and *D. chrysotoxum* (Tables S1, S2, S3 and S4). The majority of metabolite types were alkaloids, terpenoids, bibenzyls, phenanthrenes, phenolic acids, flavonoids, carboxylic acids. Figure 3A presents two heat-maps corresponding to the relative contents of different metabolites in stems and leaves of different *Dendrobium* species. As shown in Fig. 3A, alkaloids had significantly greater metabolite diversity, especially in stems and leaves of *D. nobile*, such as dendrobine, N-isopentenyl-dendrobinium, N-isopentenyl-dendroxinium, N-methyldendrobinium, homocrepidine B and dendroxine. Different content of phenanthrene, as the main metabolite, was detected in the stems of the two *Dendrobium* species. Chemometric analysis was applied to determine metabolites abundance of *D. nobile* and *D. chrysotoxum*. The results of PCA and OPLS-DA analysis showed that *D. nobile* and *D. chrysotoxum* species were clearly separated, with significant differences in chemical profiles between the two species. In addition, the metabolites in the stems and leaves of *D. nobile* differed more markedly (Fig. 3B-C). The VIP value ≥ 1.0 derived from the OPLS-DA analysis revealed the differences in metabolite profiles between *D. nobile* and *D. chrysotoxum* (Fig. 4). Notably, 18 compounds were identified as important differential metabolites between *D. nobile* and *D. chrysotoxum*, including alkaloids, phenanthrenes and terpenoids.

### Evaluations of in vitro anti-hepatoma activity

CCK-8 results showed that all four extracts could inhibit the growth of Huh-7 cells, and the anti-hepatoma activity of extracts were dose-dependent (Figure S2). According to the Table 1, among the extracts, the extract of leaves of *D. chrysotoxum* showed the strongest anti-hepatoma activity, while the stems extract of *D. nobile* showed the weakest anti-hepatoma activity. In addition, the inhibitory activity of ethanolic extract of *D. chrysanthum* leaves against HeLa human cervical cancer cells was evaluated by in vitro and in vivo assays with an IC_50_ value of 450 µg/mL [18, 40], and this result was in excellent agreement with our experimental data. As shown in Fig. S3, the Lo2 cells (normal human hepatocytes) survival rate was above 90% at all concentration gradients of *Dendrobium* samples. The results showed that all *Dendrobium* extracts with the same concentration are nontoxicity to normal cells.

**Table 1:** Anti-hepatoma activity of four extracts determined by CCK-8 (Each lower case indicates significant differences(P < 0.05))

Sample	$\bar X \pm SD$
	Anti-hepatoma activity *IC*_50_(µg/mL)
5-Fluorouracil	30.04 ± 1.16^a^
*D. Nobile-*Stem	1315.67 ± 37.50^e^
*D. Nobile-*Leaf	815.00 ± 70.73^c^
*D. chrysotoxum-*Stem	1051.67 ± 48.09^d^
*D. chrysotoxum-*Leaf	493.50 ± 28.56^b^

### Network pharmacology analysis

#### PPI network analysis

**Fig. 5:**
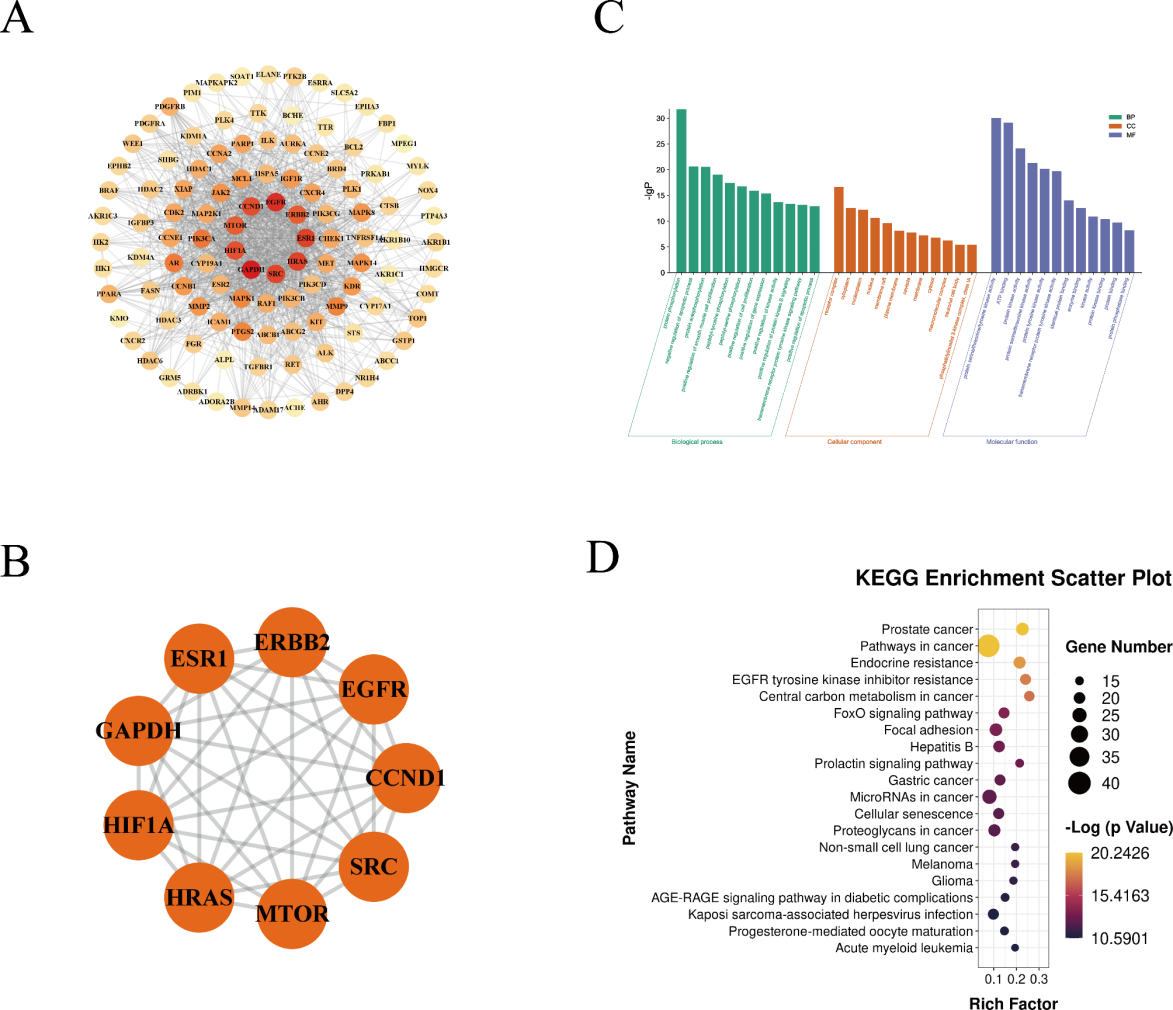
Construction of PPI network and identification of core targets. (**A**) The PPI network of potential targets was constructed. The darker the color, the higher the degree of the node; (**B**) According to the degree of the nodes, the top 9 targets were selected as the core targets. (**C**) The top 12 items in the GO biological process, cellular component and molecular function; (**D**) The top 20 items in Kyoto Encyclopedia of Genes and Genomes (KEGG) signaling pathway. (Note: The PPI network contained 112 nodes, 1192 edges, an average node degree of 21.3. Each node in the figure represents a protein, and the connections between the nodes represent the interaction between the proteins)

Because the stems and leaves of *D. chrysotoxum* have greater anti-hepatoma activity than that observed in *D. nobile*, the former species was selected. About 1526 liver cancer related targets were obtained by DisGeNET, GeneCards and OMIM. Finally, 112 potential targets for anti-hepatoma activity of *D. chrysotoxum* were obtained, the PPI network of the 112 potential targets were constructed by using the STRING database (Fig. 5A). The required interaction score was set to 0.400. Protein-protein interaction (PPI) network topological features, which have been widely used in bioinformatics to predict disease-related gene and drug targets [41]. According to the topological attribute analysis of PPI network, GAPDH, EGFR, ESR1, HRAS, SRC, CCND1, HIF1A, ERBB2 and MTOR were the protein nodes with high degree and could be considered as the core-targets of the anti-hepatoma activity of *D. chrysotoxum* (Fig. 5B).

#### Pathway enrichment analysis

DAVID is a biological information database that can provide systematic and comprehensive biological function annotation information for large-scale gene or protein lists, helping users to extract biological information from them [42]. Biological function analysis of anti-hepatoma activity of chemical components were performed by David database. The GO enrichment results for the first 12 terms of biological process, cellular component and molecular function are shown in Fig. 5C. According to statistical analysis, the results of the first 20 KEGG pathways were shown in Fig. 5D, including prostate cancer, pathways in cancer, endocrine resistance, EGFR tyrosine kinase inhibitor resistance, central carbon metabolism in cancer, FoxO signaling pathway, etc.

#### Herbs-chemicals-targets-pathways-therapeutic effects network analysis

22 components were identified by UPLC-Q-TOF-MS, 6 non-target proteins were eliminated and 454 targets were obtained by Pubchem, SwissADME and Swiss target prediction. Chrysotobibenzyl, chrysotoxin, moscatilin, gigantol and chrysotoxene were identified as core components by correlation analysis of “drug components-target”.

## Discussion

Plants are unique in the richness and diversity of their secondary metabolism, the literatures suggest that the number of metabolites produced in the plant kingdom exceeds 200,000 [43]. More and more researchers are paying attention to the study of plant secondary metabolism and pharmacological efficacy [44–48]. Although there are hundreds of millions of phytochemicals, only a small number have been isolated and identified from plants [49, 50]. The advancement of metabolomics in terms of techniques for measuring small molecule composition has enabled the rapid assay and quantification of numerous the endogenous metabolites of an organism [51]. With the optimization of the analytics platform, such as gas or liquid chromatography mass spectrometry (GC-MS and LC-MS, respectively) and nuclear magnetic resonance spectroscopy (NMR), have enabled characterizing the dynamic of metabolites [52–54]. Based on this, UPLC-Q-TOF-MS was used to identify *D. nobile* and *D. chrysotoxum* comprehensive metabolome in different tissues. Research shows that the levels of compounds from different *Dendrobium* varied greatly. In *D. nobile*, the major metabolite types were alkaloids, terpenoids, flavonoids and phenanthrenes, however, in *D. chrysotoxum*, the largest number of metabolite types belonged to bibenzyls, phenanthrenes and phenolic acids, which was consistent with previous studies [55–57]. Notably, *D. nobile* stem is abundant and diversified in alkaloids, such as dendrobine, N-isopentenyl-dendrobinium and N-methyldendrobinium, in *D. chrysotoxum*, bibenzyls and phenanthrenes secondary metabolites were significantly accumulated, such as chrysotobibenzyl, chrysotoxin, moscatilin, and chrysotoxene. Therefore, these secondary metabolites can be used as quality markers for the two *Dendrobium* species, providing a reference for species identification and quality control of *Dendrobium*. Furthermore, chemometric analysis of the metabolites differences between the two species revealed that they are quite different in evolution [58, 59]. Therefore, integrated metabolomics based on UPLC-Q-TOF-MS and chemometric analysis provides new insights for quality-oriented identification of chemical profiles of traditional chinese herbal medicines.

Modern pharmacological studies have shown that *D. nobile* and *D. chrysotoxum* has had a strong anti-cancer effect [15, 60]. For example, *D. chrysotoxum* natural products caused moderate growth delay in xenografted human hepatoma Bel7402 and melanoma A375 and induced significant vascular shutdown within 4 h of administering 100 mg/kg of the drug [61]. In addition, *D. nobile* extracts down regulated the expression level of decoy receptor-3 and synergized with Fas ligand to bring about apoptotic cell death in pancreatic adenocarcinoma cells [62]. However, the inhibition rates of the two dendrobium species on hepatocellular carcinoma cells were different. The study showed that the anti-hepatoma effect of *D. chrysotoxum* was significantly better than that of *D. nobile*, and it was worth noting that *D. chrysotoxum* leaves had the strong anti-hepatoma effect. Due to the wide variation in the types and contents of secondary metabolites of different *Dendrobium* species, the differences in their active ingredients may lead to the differences in their pharmacological activities.

The result of KEGG enrichment indicated that the 112 potential targets were highly enriched in the EGFR tyrosine kinase inhibitor resistance and FoX O signaling pathway. It has been confirmed that EGFR targets have a significant impact on the proliferation and migration of liver cancer cells [63, 64], which is consistent with the results of network pharmacological analysis in this study. In addition, *FoX O* signaling pathway related to a variety of tumors. The research of Yang et al. and Wang et al. indicated that *FOXO1* is weakly expressed in liver cancer tissue, which results in abnormal cell proliferation and cell apoptosis [65, 66]. The influence of *D. chrysotoxum* on hepatocellular carcinoma inhibition may depend on making an impact on EGFR tyrosine kinase inhibitor resistance pathway and *FoX O* signaling pathway.

The stems of *D. nobile* and *D. chrysotoxum* had a long history of medicinal use, but the leaves are often discarded during the production of medicinal materials. And the leaves on the *Dendrobium* stems are sometimes peeled for aesthetic reasons. However, the metabolite fingerprint results here indicated that there were still a large number of bioactive compounds in the leaves of *D. nobile* and *D. chrysotoxum*. Previous pharmacological studies also have shown that have also shown that *Dendrobium* leaves play an important role in dermatologic disorders, metabolic syndromes, nervous system disorders, and musculoskeletal system disorders [18]. Therefore, it is necessary to develop an approach for the secondary utilization of leaves. Moreover, comprehensive metabolomics results showed that leaves of *D. nobile* and *D. chrysotoxum* were rich in many bioactive components and had excellent pharmacological effects (Fig. 3; Table 1), indicating that the leaves should be preserved as much as possible in the process of crude drug production. However, in traditional processing of *Dendrobiums*, the leaves were removed after thoroughly washing. Therefore, *Dendrobium* leaves show potential for the research and development of pharmacological biomolecules.

## Conclusions

In this study, mass spectrometry-based metabolomic and multivariate statistical analysis were conducted to screen the differential metabolites in *D. nobile* and *D. chrysotoxum*. We screened differential ions in the positive-ion and negative-ion model of UPLC-Q-TOF-MS/MS using OPLS-DA and PCA. CCK-8 results showed that *D. nobile* and *D. chrysotoxum* extracts could inhibit the growth of Huh-7 cells, and the anti-hepatoma activity of extracts were dose-dependent. Network pharmacology analysis revealed chrysotobibenzyl, chrysotoxin, moscatilin, gigantol and chrysotoxene as relevant compounds for *D. chrysotoxum* anti-hepatoma activity. Our research provided a effective method for rapid screening and identification of the differential metabolites in different *Dendrobium* species, and provided candidate chemical markers for herb quality screening of *D. nobile* and *D. chrysotoxum*.

## Electronic supplementary material

Below is the link to the electronic supplementary material.

Supplementary Material 1

Supplementary Material 2: Review history.

## Data Availability

The datasets used and/or analyzed during the current study are available from the corresponding author on request.

## Declarations

### Review history

The review history is available as Supplementary Material 2.
