# Supplementary material for: Comprehensive chemical profiling of two Dendrobium species and identification of anti-hepatoma active constituents from Dendrobium chrysotoxum by network pharmacology
Source: BMC Complement Med Ther. 2023 Jul 1;23:217. doi: 10.1186/s12906-023-04048-y (PMC10314590; doi:10.1186/s12906-023-04048-y)
Supplement: Supplementary file 1 — Supplementary Material 1 [file 12906_2023_4048_MOESM1_ESM.doc]

**Supplementary material**

**Comprehensive chemical profiling of two *Dendrobium* species and identification of anti-hepatoma active constituents from *Dendrobium chrysotoxum* by** **Network Pharmacology**

**
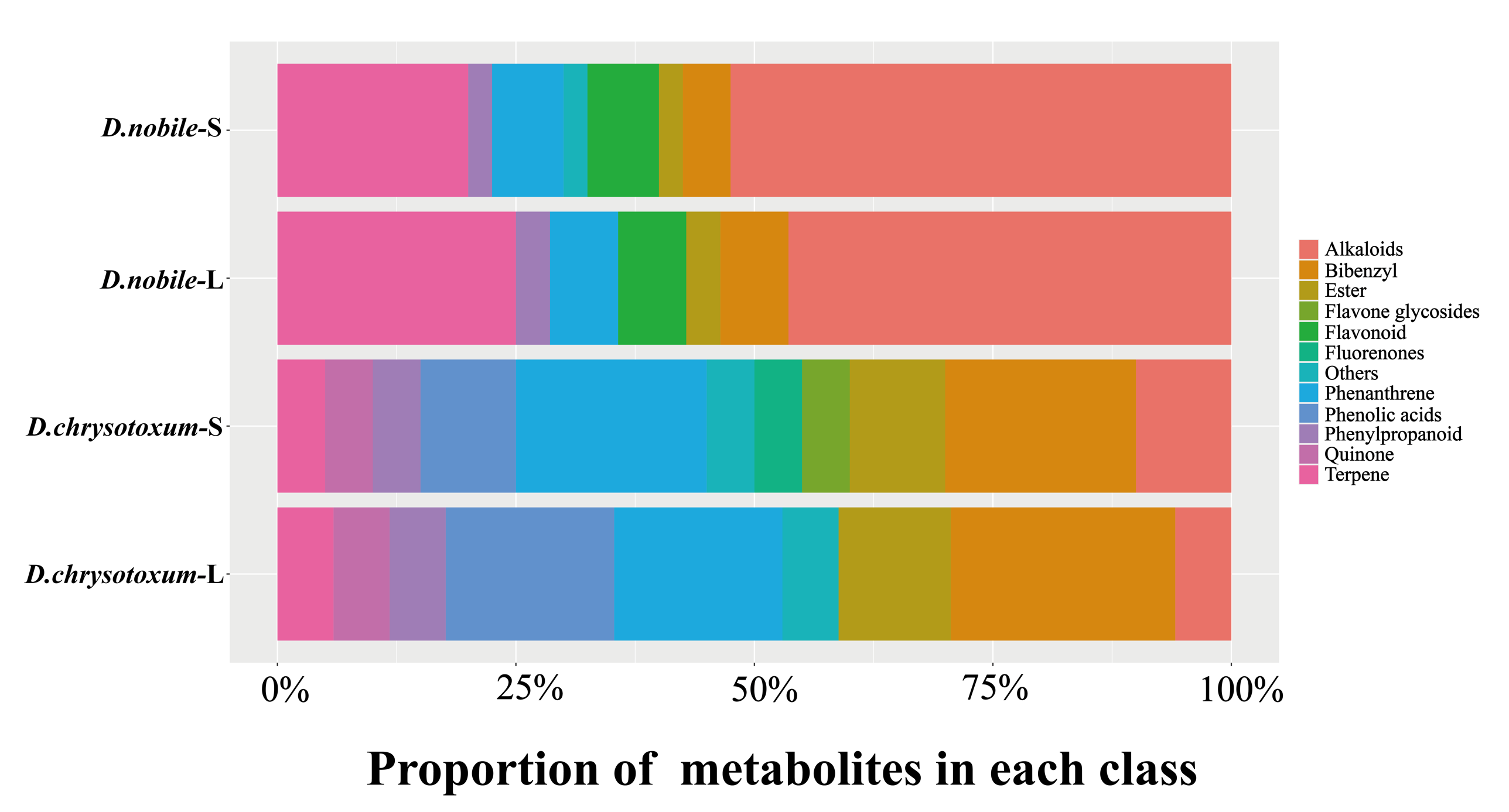
**

**Fig. S1.** Proportions of metabolites in each samples.


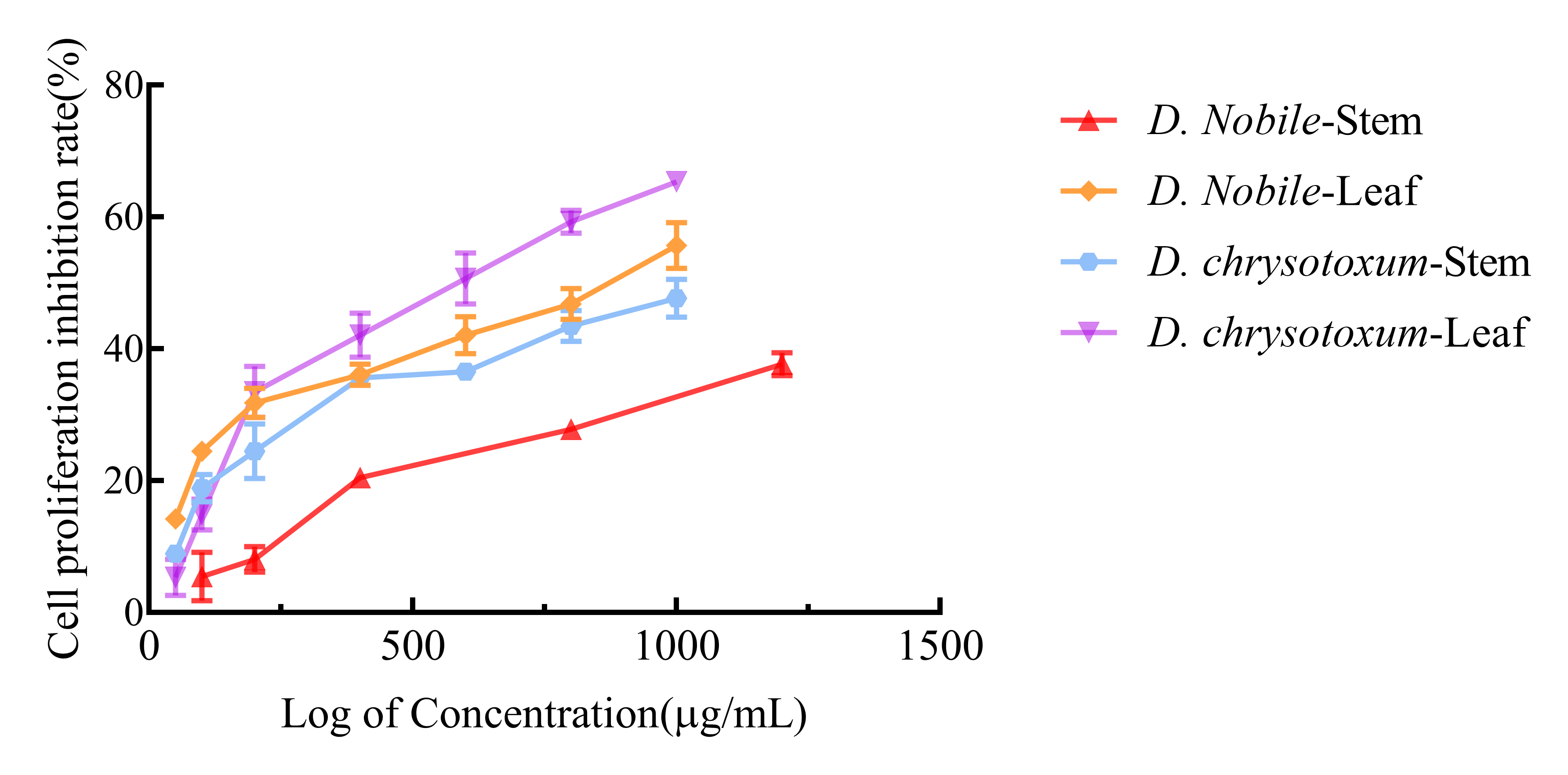


**Fig. S2.** Cell proliferation inhibition rate.


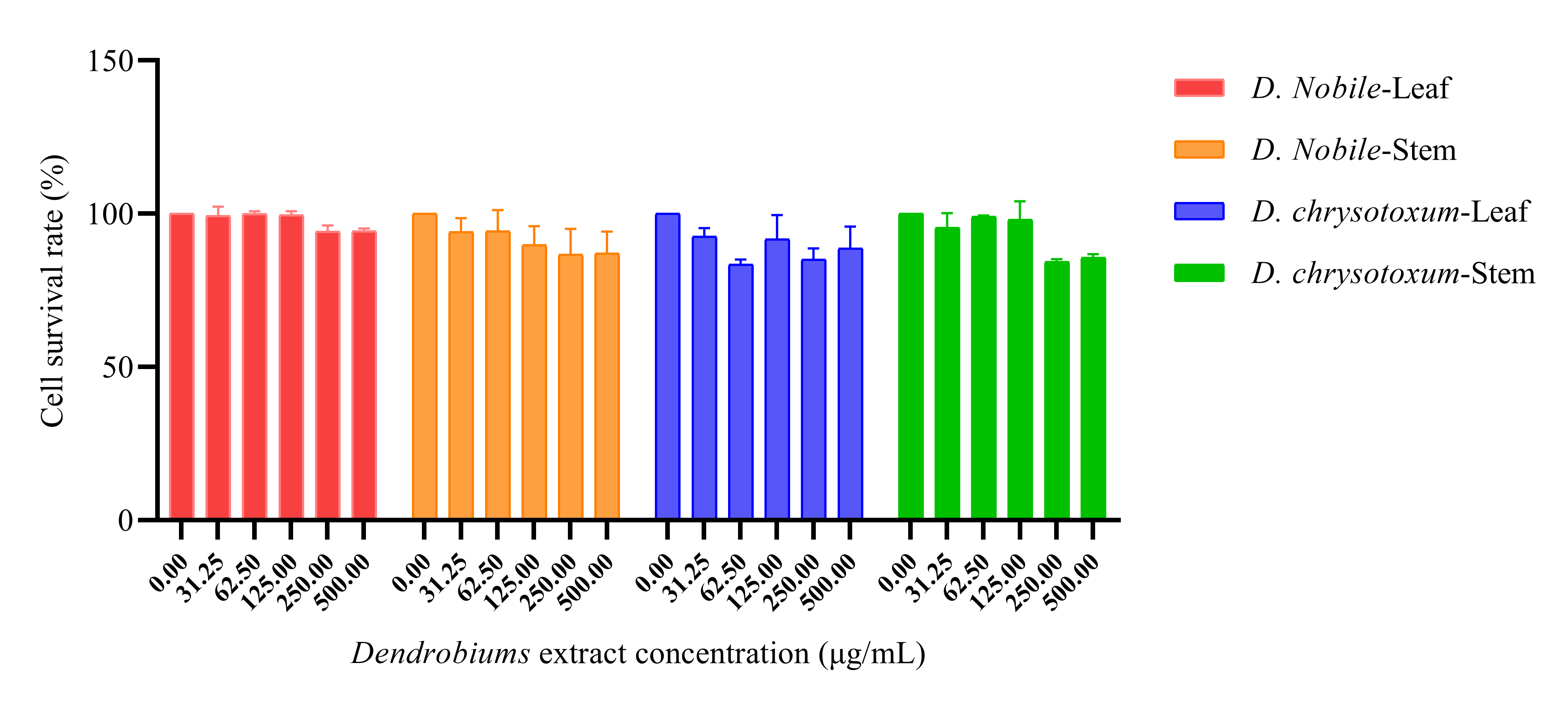


**Fig. S3.** The results of cytotoxicity ass

**Table S1.** Characterization of major metabolites in the stems of *D. nobile* by LC-MS/MS.

| No. | Compound Name | tR(Min) | Adduct Ion | Theoretical m/z | Measured m/z | Molecular Fomular | Classification |
| --- | --- | --- | --- | --- | --- | --- | --- |
|
| 1 | Dendrobine isomer1 | 6.60 | [M+H]+ | 264.1964 | 264.1982 | C16H25O2N | Alkaloids |
| 2 | Dendrobine isomer2 | 7.35 | [M+H]+ | 264.1964 | 264.1982 | C16H25O2N | Alkaloids |
| 3 | N-isopentenyl-dendrobinium | 10.16 | [M+H]+ | 332.2590 | 332.2524 | C16H25O2N | Alkaloids |
| 4 | Nobilonine | 7.80 | [M+H]+ | 294.2069 | 294.1932 | C17H27O3N | Alkaloids |
| 5 | N-isopentenyl-dendroxinium | 10.76 | [M]+ | 360.2539 | 360.2463 | C22H33NO3 | Alkaloids |
| 6 | N-methyldendrobinium | 6.53 | [M+H]+ | 279.2198 | 279.2190 | C17H28O2N | Alkaloids |
| 7 | Mubironine B | 6.39 | [M+H]+ | 250.1807 | 250.1825 | C15H23O2N | Alkaloids |
| 8 | Dendroamine | 5.26 | [M+H]+ | 280.1913 | 280.1908 | C16H25O3N | Alkaloids |
| 9 | 6-hydroxynobiline | 5.95 | [M+H]+ | 310.2018 | 310.2023 | C17H27O4N | Alkaloids |
| 10 | N-isopentenyl-6-hydroxy-dendroxinium | 9.66 | [M]+ | 376.2488 | 376.2523 | C22H34O4N | Alkaloids |
| 11 | Homocrepidine B | 10.73 | [M+H]+ | 291.2198 | 291.1807 | C18H28O2N | Alkaloids |
| 12 | dendrine | 8.42 | [M+H]+ | 336.2175 | 336.218 | C19H29NO4 | Alkaloids |
| 13 | 8-hydroxy-dendroxine | 4.23 | [M+H]+ | 308.1862 | 308.184 | C17H25NO4 | Alkaloids |
| 14 | dendroxine | 10.69 | [M+H]+ | 292.1913 | 292.1975 | C17H25NO3 | Alkaloids |
| 15 | 2-hydroxydendrobine/6-hydroxydendrobine | 5.04 | [M+H]+ | 280.1913 | 280.1908 | C16H25O3N | Alkaloids |
| 16 | adenosine | 2.51 | [M+H]+ | 268.1046 | 268.1041 | C10H13N5O4 | Alkaloids |
| 17 | N-trans-feruloyl tyramine | 4.12 | [M+H]+ | 314.1392 | 314.1608 | C18H19NO4 | Alkaloids |
| 18 | dendronobiline A | 4.85 | [M+H]+ | 320.2226 | 320.1877 | C19H29NO3 | Alkaloids |
| 19 | succinylcarnitine | 6.29 | [M+H]+ | 262.1290 | 262.1561 | C11H19NO6 | Alkaloids |
| 20 | Crepidine | 3.61 | [M+H]+ | 344.2226 | 344.2299 | C21H29NO3 | Alkaloids |
| 21 | N-trans-p-coumaroyltyramine | 9.73 | [M-H]- | 282.1130 | 282.2518 | C17H17O3N | Alkaloids |
| 22 | crepidatin/chrysotoxin | 3.13 | [M+H]+ | 319.1546 | 319.1196 | C18H22O5 | Bibenzyl |
| 23 | pinoresinol | 0.69 | [M+Na]+ | 381.1314 | 381.0818 | C20H22O6 | Bibenzyl |
| 24 | denobilone B | 3.06 | [M+H]+ | 261.1127 | 261.1315 | C15H16O4 | phenanthrene |
| 25 | denobilone C | 1.51 | [M+H]+ | 275.0920 | 275.111 | C16H18O4 | phenanthrene |
| 26 | flavanthridin | 7.80 | [M+Na]+ | 295.0946 | 295.2120 | C15H12O5 | phenanthrene |
| 27 | 2,4,7-Trihydroxy-9,10-dihydrophenanthrene | 8.70 | [M-H]- | 227.0708 | 227.1992 | C14H12O3 | phenanthrene |
| 28 | dendronobilin C/dendronobilin J | 4.64 | [M]+ | 298.1416 | 298.1664 | C15H22O6 | Terpene |
| 29 | dendronobilin L/dendronobilin M | 4.71 | [M+Na]+ | 323.1471 | 323.2043 | C15H24O6 | Terpene |
| 30 | dendronobilin B/dendronobilin D/dendronobilin E | 8.63 | [M+Na]+ | 307.1521 | 307.2114 | C15H24O5 | Terpene |
| 31 | dendronobilin F | 3.37 | [M+H]+ | 305.1389 | 305.1528 | C17H20O5 | Terpene |
| 32 | dendroterpene C | 7.08 | [M+H]+ | 265.1440 | 265.1765 | C15H20O4 | Terpene |
| 33 | dendroside G | 3.78 | [M+H]+ | 469.2050 | 469.1225 | C21H34O10 | Terpene |
| 34 | dendronobilin H | 1.27 | [M+H]+ | 277.1780 | 277.1476 | C15H26O3 | Terpene |
| 35 | Dendronobiloside C/D | 5.74 | [M+H]+ | 561.2911 | 561.3824 | C27H44O12 | Terpene |
| 36 | Apigenin | 9.90 | [M-H]- | 269.0450 | 269.2495 | C15H10O5 | Flavonoid |
| 37 | Naringenin | 6.46 | [M-H]- | 271.0607 | 271.0598 | C15H12O5 | Flavonoid |
| 38 | isoliquiritin | 3.75 | [M+Na]+ | 441.1162 | 441.1372 | C21H22O9 | Flavonoid |
| 39 | medioresinol | 3.40 | [M+H]+ | 389.1600 | 389.1806 | C21H24O7 | Phenylpropanoid |
| 40 | dhydroconiferyl dihydro-p-cumarate | 5.95 | [M-H]- | 329.1389 | 329.2319 | C19H22O5 | Ester |
| 41 | 4-(2-(2",2"-dimethyl-6"-methy lene-4”-oxocyclohexyl) vi-nyl) furan-2(5H)-one | 1.92 | [M]+ | 246.1256 | 246.1342 | C15H18O3 | Others |

**Table S2.** Characterization of major metabolites in the leaf of *D. nobile* by LC-MS/MS.

| No. | Compound Name | tR(Min) | Adduct Ion | Theoretical m/z | Measured m/z | Molecular Fomular | Classification |
| --- | --- | --- | --- | --- | --- | --- | --- |
|
| 1 | Dendrobine isomer1 | 6.60 | [M+H]+ | 264.1964 | 264.1982 | C16H25O2N | Alkaloids |
| 2 | Dendrobine isomer2 | 7.35 | [M+H]+ | 264.1964 | 264.1982 | C16H25O2N | Alkaloids |
| 3 | N-isopentenyl-dendrobinium | 10.12 | [M+H]+ | 332.2590 | 332.2524 | C16H25O2N | Alkaloids |
| 4 | N-methyldendrobinium | 6.43 | [M+H]+ | 279.2198 | 279.2155 | C17H28O2N | Alkaloids |
| 5 | Mubironine B | 6.32 | [M+H]+ | 250.1807 | 250.1825 | C15H23O2N | Alkaloids |
| 6 | Dendroamine | 5.55 | [M+H]+ | 280.1913 | 280.1976 | C16H25O3N | Alkaloids |
| 7 | dendrine | 8.46 | [M+H]+ | 336.2175 | 336.218 | C19H29NO4 | Alkaloids |
| 8 | 8-hydroxy-dendroxine | 4.26 | [M+H]+ | 308.1862 | 308.1876 | C17H25NO4 | Alkaloids |
| 9 | adenosine | 2.47 | [M+H]+ | 268.1046 | 268.1041 | C10H13N5O4 | Alkaloids |
| 10 | succinylcarnitine | 6.12 | [M+H]+ | 262.1290 | 262.1561 | C11H19NO6 | Alkaloids |
| 11 | dendronobiline A | 7.91 | [M+H]+ | 320.2226 | 320.2243 | C19H29NO3 | Alkaloids |
| 12 | Crepidine | 3.61 | [M+H]+ | 344.2226 | 344.2299 | C21H29NO3 | Alkaloids |
| 13 | N-trans**-***p***-**coumaroyltyramine | 9.9 | [M-H]- | 282.1130 | 282.2518 | C17H17O3N | Alkaloids |
| 14 | crepidatin/chrysotoxin | 3.13 | [M+H]+ | 319.1546 | 319.1196 | C18H22O5 | Bibenzyl |
| 15 | pinoresinol | 0.69 | [M+Na]+ | 381.1314 | 381.0818 | C20H22O6 | Bibenzyl |
| 16 | denobilone C | 1.47 | [M+H]+ | 275.1283 | 275.111 | C16H18O4 | phenanthrene |
| 17 | erianthridin | 1.44 | [M+H]+ | 273.1127 | 273.0955 | C16H16O4 | phenanthrene |
| 18 | dendronobilin B/dendronobilin D/dendronobilin E | 8.28 | [M+Na]+ | 307.1521 | 307.2114 | C15H24O5 | Terpene |
| 19 | dendroterpene C | 6.80 | [M+H]+ | 265.1440 | 265.1765 | C15H20O4 | Terpene |
| 21 | dendronobilin H | 1.23 | [M+H]+ | 277.1780 | 277.1476 | C15H26O3 | Terpene |
| 22 | dendroside B | 5.33 | [M+H]+ | 581.2234 | 581.1702 | C27H46O13 | Terpene |
| 23 | dendroside D isom1 | 11.28 | [M+H]+ | 593.2809 | 593.2755 | C27H44O14 | Terpene |
| 24 | dendroside D isom2 | 11.55 | [M+H]+ | 593.2809 | 593.2755 | C27H44O14 | Terpene |
| 25 | isoliquiritin | 3.75 | [M+Na]+ | 441.1162 | 441.1372 | C21H22O9 | Flavonoid |
| 26 | Apigenin | 10.1 | [M-H]- | 269.0450 | 269.2495 | C15H10O5 | Flavonoid |
| 27 | medioresinol | 3.4 | [M+H]+ | 389.1600 | 389.1806 | C21H24O7 | Phenylpropanoid |
| 28 | dhydroconiferyl dihydro*-p*-cumarate | 5.93 | [M-H]- | 329.1389 | 329.2319 | C19H22O5 | Ester |

**Table S3.** Characterization of major metabolites in the stems of *D. chrysotoxum* by LC-MS/MS.

| No. | Compound Name | tR(Min) | Adduct Ion | Theoretical m/z | Measured m/z | Molecular Fomular | Classification |
| --- | --- | --- | --- | --- | --- | --- | --- |
|
| 1 | Dendrobine | 7.53 | [M+H]+ | 264.1964 | 264.1954 | C16H25O2N | Alkaloids |
| 2 | adenosine | 2.47 | [M+H]+ | 268.1046 | 268.1041 | C10H13N5O4 | Alkaloids |
| 3 | chrysotobibenzyl | 8.64 | [M+H]+ | 333.1702 | 333.2047 | C19H24O5 | Bibenzyl |
| 4 | moscatilin | 9.42 | [M+Na]+ | 327.1208 | 327.0781 | C17H20O5 | Bibenzyl |
| 5 | gigantol | 13.61 | [M]+ | 274.1205 | 274.2734 | C16H18O4 | Bibenzyl |
| 6 | chrysotoxin/erianin | 8.22 | [M+H]+ | 319.1546 | 319.1561 | C18H22O5 | Bibenzyl |
| 7 | 2, 7-dihydroxy- 3, 4, 6- trimethoxy- phenanthrene | 2.58 | [M]+ | 300.0998 | 300.1446 | C17H16O5 | Phenanthrene |
| 8 | chrysotoxene | 2.34 | [M]+ | 314.1154 | 314.0919 | C18H18O5 | Phenanthrene |
| 9 | Confusarin | 10.14 | [M+H]+ | 301.1076 | 301.1415 | C17H16O5 | Phenanthrene |
| 10 | 2,7-dihydroxy-3,4-dimethoxyphenanthrene | 11.93 | [M]+ | 270.0892 | 270.2794 | C16H14O4 | Phenanthrene |
| 11 | defuscin | 13.48 | [M+Na]+ | 607.5066 | 607.2914 | C39H68O3 | Ester |
| 12 | 3-(4-hydroxy-3-methoxy-phenyl)-acrylic acid octacosyl ester | 5.74 | [M]+ | 586.4961 | 586.4180 | C38H66O4 | Ester |
| 13 | dengibsin | 8.63 | [M]+ | 242.0579 | 242.1540 | C14H10O4 | Fluorenones |
| 14 | Emodin/aloe-Emodin | 9.97 | [M-H]- | 269.0450 | 269.2462 | C15H10O5 | Quinone |
| 15 | episyringaresinol | 3.98 | [M+Na]+ | 441.1525 | 441.1759 | C22H26O8 | Phenylpropanoid |
| 16 | Erigeside I I | 5.40 | [M+Na]+ | 379.1369 | 379.1391 | C17H24O8 | Flavone glycosides |
| 17 | 3,4-dimethoxybenzoic acid | 0.96 | [M-H]- | 181.0501 | 181.0707 | C9H9O4 | Phenolic acids |
| 18 | 5-hydroxy-6-methoxyphenylethanol | 6.29 | [M]+ | 169.0865 | 169.0787 | C9H12O3 | Phenolic acids |
| 19 | flakinins B | 7.77 | [M-H]- | 295.1546 | 295.2265 | C16H24O5 | Terpene |
| 20 | β-Sitosterol | 8.70 | [M+H]+ | 415.3940 | 415.2127 | C29H50O | Others |

**Table S4.** Characterization of major metabolites in the leaf of *D. chrysotoxum* by LC-MS/MS.

| No. | Compound Name | tR(Min) | Adduct Ion | Theoretical m/z | Measured m/z | Molecular Fomular | Classification |
| --- | --- | --- | --- | --- | --- | --- | --- |
|
| 1 | Dendrobine | 7.53 | [M+H]+ | 264.1964 | 264.1954 | C16H25O2N | Alkaloids |
| 2 | chrysotobibenzyl | 8.64 | [M+H]+ | 333.1702 | 333.2047 | C19H24O5 | Bibenzyl |
| 3 | moscatilin | 9.42 | [M+Na]+ | 327.1208 | 327.0781 | C17H20O5 | Bibenzyl |
| 4 | gigantol | 13.61 | [M]+ | 274.1205 | 274.2734 | C16H18O4 | Bibenzyl |
| 5 | chrysotoxin/erianin | 8.22 | [M+H]+ | 319.1546 | 319.1561 | C18H22O5 | Bibenzyl |
| 6 | chrysotoxene | 2.34 | [M]+ | 314.1154 | 314.0919 | C18H18O5 | Phenanthrene |
| 7 | Confusarin | 10.14 | [M+H]+ | 301.1076 | 301.1415 | C17H16O5 | Phenanthrene |
| 8 | 2,7-dihydroxy-3,4-dimethoxyphenanthrene | 11.93 | [M]+ | 270.0892 | 270.2794 | C16H14O4 | Phenanthrene |
| 9 | defuscin | 13.48 | [M+Na]+ | 607.5066 | 607.2914 | C39H68O3 | Ester |
| 10 | 3-(4-hydroxy-3-methoxy-phenyl)-acrylic acid octacosyl ester | 5.74 | [M]+ | 586.4961 | 586.4180 | C38H66O4 | Ester |
| 11 | Emodin/aloe-Emodin | 9.97 | [M-H]- | 269.0450 | 269.2462 | C15H10O5 | Quinone |
| 12 | p-hydroxyphenylpropanoic acid | 1.96 | [M-H]- | 165.0552 | 165.0718 | C9H10O3 | Phenylpropanoid |
| 13 | dendroflorin | 0.72 | [M]+ | 258.0528 | 258.1314 | C14H10O5 | Phenolic acids |
| 14 | 3,4-dimethoxybenzoic acid | 0.96 | [M-H]- | 181.0501 | 181.0707 | C9H9O4 | Phenolic acids |
| 15 | 5-hydroxy-6-methoxyphenylethanol | 6.29 | [M]+ | 169.0865 | 169.0787 | C9H12O3 | Phenolic acids |
| 16 | flakinins B | 7.91 | [M-H]- | 295.1546 | 295.2265 | C16H24O5 | Terpene |
| 17 | β-Sitosterol | 8.70 | [M+H]+ | 415.3940 | 415.2127 | C29H50O | Others |
