# Supplementary material for: Comprehensive chemical profiling of two Dendrobium species and identification of anti-hepatoma active constituents from Dendrobium chrysotoxum by network pharmacology
Source: BMC Complement Med Ther. 2023 Jul 1;23:217. doi: 10.1186/s12906-023-04048-y (PMC10314590; doi:10.1186/s12906-023-04048-y)
Supplement: Supplementary file 2 — Supplementary Material 2: Review history. [file 12906_2023_4048_MOESM2_ESM.docx]

MS ID: <https://editorial.nature.com/submissions/9c007b84-b822-452e-a68f-886aab82694d/reviews-and-decisions/versions/1.2>

**Comprehensive chemical profiling of two *Dendrobium* species and identification of anti-hepatoma active constituents from *Dendrobium chrysotoxum* by Network Pharmacology**

**Peer-Review**

V1

**Reviewer 1** 14-Nov-22

**Feedback for author(s)**

Introduction

Line 24: “favonoids” should be replaced by flavonoids

Line 26-27: “Plants………….medicinal effects” Please mention some important medicinal effects.

Materials and methods

All the procedures have been written in detail and briefly. However, some points are missed, for example, at what stage did the authors collect samples for LC-MS analysis? Please provide citations for the methods that they followed for sample preparation, extraction etc.

The utilization of advanced softwares for analysis is highly appreciated.

Results

Results displayed a great diversity among different metabolites in D. nobile stem and leaf. However, figures are not visible clearly. Please provide high quality images in TIF format

Under the sub-heading, “evaluations of in vitro anti-hepatoma activity”, the table depicts that D. Nobile-stem exhibited highest anti-hepatoma activity which is contradictory to what is written in the text portion that the “stem extract of D. nobile showed the weakest anti-hepatoma activity”.

The authors have performed an outstanding work displaying few interesting novel findings. It is an interesting finding that there is no significant difference in the chemical composition of stems and leaves of Dendrobium species. Another good finding is that leaves which were traditionally removed from plants showed better bioactive constituents in comparison to stem.

**Feedback for editor**

```
Your recommendation
	Revise

Is the study design appropriate to answer the research question (including the use of appropriate controls), and are the conclusions supported by the evidence presented?
	Yes

Are the methods sufficiently described to allow the study to be repeated?
	Yes

Is the use of statistics and treatment of uncertainties appropriate?
	Yes

Is the presentation of the work clear?
	Yes

Are the images in this manuscript (including electrophoretic gels and blots) free from apparent manipulation?
	Yes

Confidential Comments to the Editor
minor revision
```

**Reviewer 2** 4-Nov-22

**Feedback for author(s)**

Authors have attempted to describe the chemical profiling of two Dendrobium species and identification of anti-hepatoma active constituents from Dendrobium chrysotoxum by Network Pharmacology. Through the compound-target-pathway network, nine targets were found to be considered as core-targets of anti-hepatoma activity. However, the manuscript needs major revision in many sections and different aspects to publishing in BMC PLANT BIOLOGY. For the manuscript "Comprehensive chemical profiling of two Dendrobium species and identification of anti-hepatoma active constituents from Dendrobium chrysotoxum by Network Pharmacology", I suggest the following things.

Major things：

1. There are so many errors in English, one gets distracted while reading because of language issues. The author should check the English throughout the manuscript with help of the native speaker.

2. The abstract part lacks the flow in the narration of importance of the research, and the results and conclusions contain many redundant statements which should be omitted or revised.

3. Fig. 1A would be better to replace with a more distinct figure which exhibits the unique feature of the stem of D. chrysotoxum.

4. Fig. 1D is not obtained from this research, and is not required.

5. The authors must provide clearer information on tissues used in the research since the growth year of stems, as well as collect time have great influence on chemical profiling.

6. Why do you need to have both Figures 2B and 2C? In my opinion, 2C represent the same story with 2B. Isn't it? I suggest you select 2C and then include the other in the supplement.

7. At least 3-5 predominant constituents should be detected to confirm the reliability of metabolome.

8. For Fig. 3A, what do the “-S”, “-S1” mean should be explained in the legend. And for Fig. 3B and 3C, labels in figures should be matched with each specific sample. I cannot agree with the statement in line 259, the PCA result indicated that only tissues in D. nobile can be clear separate through metabolites analysis.

9. Which cell line were you used as control in evaluation of anti-cancer test? Were the extracts toxic to normal cells? What were you used as solvent for the extracts? Solvent should also be used as control. Because IC50 of 493.50 μg/mL is really a lot to me even if the extracts are mix compound. For the result “the anti-hepatoma activity of extracts were dose-dependent”, please provide proves.

10. It is really hard to read this diagram in its current state, I suggest the authors should edit this figure to highlight related proteins (such as change the node size based on node degree?) and further improve the readability.

11. Network analysis is routine narrative and authors need to look into the results again and dig deeper for the results.

12. Discussion is the weakest part of the manuscript, this section should be rewritten and reconsidered totally.

Minor things：

Line 20-21: Please use proper words and mind the tense.

Line 56-58: Please correct this sentence in English.

Line 61: medicinal plant-based → medicinal plant.

Line 62: ailments → disease; “as safer and more effective”, please correct this sentence in English.

Line 63: present → presented.

Line 66-67: Please correct this sentence in English.

Line 69: The edition of Chinese Pharmacopoeia should be specified.

Line 82-83: Redundant statement.

Line 95: Chinese.

Line 226-229: Statement not required.

Line 231: Remove “types”.

Line 249-250: Redundant statement.

Line 250-252: Statement not required.

Line 282: leaf → leaves. Actually, they did not show excellent anti-hepatoma effect in this research.

Line 287-290: The statement should be removed to figure legend.

**Feedback for editor**

```
Your recommendation
	Revise

Is the study design appropriate to answer the research question (including the use of appropriate controls), and are the conclusions supported by the evidence presented?
	No, but these points can be addressed with revisions
	Comments: I think some  more experiments should be conducted to support their conclusions.

Are the methods sufficiently described to allow the study to be repeated?
	Yes

Is the use of statistics and treatment of uncertainties appropriate?
	Yes

Is the presentation of the work clear?
	No, it needs some language corrections before being published
	Comments: There are so many errors in English, one gets distracted while reading because of language issues.

Are the images in this manuscript (including electrophoretic gels and blots) free from apparent manipulation?
	Yes

Confidential Comments to the Editor
There's no ethical concern nor conflit of interest reparding the experiments .
```

V2

**Reviewer 2** 29-Dec-22

**Feedback for author(s)**

1. There are still some apparent errors in English such as Line 20-21, the author should double-check the English throughout the manuscript.

2. As for the result “Evaluations of in vitro anti-hepatoma activity”, the anti-cancer test was conducted in cells cultured in vitro. However, even a high concentration of NaCl solution will lead to cell death in anti-cancer tests in vitro. You need to prove that the Dendrobium extracts with the same concentration are nontoxicity to normal cells.

**Feedback for editor**

```
Your recommendation
	Revise

Is the study design appropriate to answer the research question (including the use of appropriate controls), and are the conclusions supported by the evidence presented?
	No, but these points can be addressed with revisions
	Comments: Control experiment is  needed to prove the anti-hepatoma activity of the Dendrobium extracts.

Are the methods sufficiently described to allow the study to be repeated?
	Yes

Is the use of statistics and treatment of uncertainties appropriate?
	Yes

Is the presentation of the work clear?
	No, it needs some language corrections before being published
	Comments: There are still some apparent errors in English.

Are the images in this manuscript (including electrophoretic gels and blots) free from apparent manipulation?
	Yes

Confidential Comments to the Editor
```

**Reviewer 1** 18-Dec-22

**Feedback for author(s)**

Authors have addressed concerns raised, and modified the manuscript accordingly. It may be accepted for publication.

**Feedback for editor**

```
Your recommendation
	Accept

Is the study design appropriate to answer the research question (including the use of appropriate controls), and are the conclusions supported by the evidence presented?
	Yes

Are the methods sufficiently described to allow the study to be repeated?
	Yes

Is the use of statistics and treatment of uncertainties appropriate?
	Yes

Is the presentation of the work clear?
	Yes

Are the images in this manuscript (including electrophoretic gels and blots) free from apparent manipulation?
	Yes

Confidential Comments to the Editor
paper may be accepted
```

V3

**Reviewer 2** 15-Feb-23

**Feedback for author(s)**

The concerns that we proposed are properly addressed. Now I'm sure that this revised manuscript can be accepted for publication in BMC Plant Biology.

**Feedback for editor**

```
Your recommendation
	Accept

Is the study design appropriate to answer the research question (including the use of appropriate controls), and are the conclusions supported by the evidence presented?
	Yes

Are the methods sufficiently described to allow the study to be repeated?
	Yes

Is the use of statistics and treatment of uncertainties appropriate?
	Yes

Is the presentation of the work clear?
	Yes

Are the images in this manuscript (including electrophoretic gels and blots) free from apparent manipulation?
	Yes

Confidential Comments to the Editor
```

**Editor’s Comments**

#### **Revision requested** | **16-Feb-23**

```
Editor’s Comments:

The authors have addressed the concerns raised by reviewers and modified the manuscript accordingly. The manuscript has improved, but I believe there are still issues that must be addressed to improve its overall quality.

Overall, the manuscript still shows many grammatical errors and problematic sentences. The authors should carefully check and edit the language, and possibly they should seek support from a professional language editing service. Moreover, redundant and unclear statements should be changed. Below I provide many examples that must be corrected; however, please carefully screen the entire manuscript to improve the message and language.

1)	Line 20: The sentence should be rephrased as: ‘Dendrobium nobile and Dendrobium chrysotoxum are important species…’.
2)	Line 21: ‘The material basis for the medicinal effects of these two plants is still unknown, and…’; what do the authors mean by ‘material basis’? Possibly I would rephrase as: ‘However, the medicinal properties of these two plants remain poorly understood.’
3)	Line 22: the sentence ‘and the biomarkers associated with anticancer are not entirely understood at present.’ should be deleted, because it is too specific for the context here.
4)	Lines 23-26 should be rephrased as or similar to: ‘This study focused on the comprehensive chemical profiling of D. nobile and D. chrysotoxum, and identification of active compounds and predictive targets for anti-hepatoma activity in D. chrysotoxum extracts by Network Pharmacology.’ Is this in line with authors’ message?
5)	Line 34: please rephrase as: ‘In order to find the potential mechanism of anti-hepatoma activity of D. chrysotoxum,’.
6)	Lines 59-60: the sentence is incomplete; please correct.
7)	Line 77: ‘chemical compounds’
8)	Lines 79-81: ‘Chinese medicine’ should be removed here, as this is a general statement and the application of the approach to TCM compounds should be stressed later (see below). Rephrase as or similar to: ‘In recent years, network pharmacological analysis has been effectively applied to predict the relationship between protein targets, active compounds and related diseases.’
9)	Lines 82-84 should be changed into: ‘This approach attempts to explore disease-related, multiple active compounds and targets, thus allowing to characterize the multi-target mechanism of bioactive compounds [23-24]’ or similar. Traditional Chinese Medicine (TCM) is introduced later and should not be here.
10)	Lines 84-87 are redundant with sentences above: ‘The core of network pharmacology is to construct a “bioactive compound-target-disease” relationship network, and then predict the  mechanism of the drug action on the organism through the network relationship of a holistic approach.’. Please remove.
11)	Lines 87-89: please rephrase as or similar to ‘This analysis can provide insights into the link between Traditional Chinese Medicine (TCM) bioactive compounds and diseases, possibly revealing the pharmacological properties of multiple biomolecules found in medicinal plants [25].’
12)	Line 92: comprehensive metabolite profiling
13)	IMPORTANT NOTE: the authors must clarify that they used only the Huh-7 cell line in this study and no other human samples; this should be clearly stated in Materials and Method, and explained in Ethics approval. Moreover, the authors should state where the cell line was purchased and provide the seller details, or provide details on how the cells were obtained in Materials and Methods (see below).
14)	In line with the aspect above, please modify/amend the following sentence (lines 95-96) as or similar to ‘A network pharmacology approach was applied to characterize the possible mechanism of action of D. chrysotoxum compounds on the human hepatoma Huh-7 cell line’
15)	Line 124-125: please rephrase as ‘The human hepatoma Huh7 cell line used in this study was donated by Dr Li Gongchu's research group of Zhejiang Sci-Tech University (Hangzhou, China)’; and this part should be mentioned in the section 2.4 (subsection should be ‘Cell Cultures and CCK-8 assay of plant extracts). Please, specify in Ethics approval that only Huh7 cell line was used.
16)	Line 160: please rephrase as ‘Human hepatoma Huh-7 cells were maintained routinely in…’.
17)	Line 99: ‘while the potential targets and signalling pathways were…’
18)	Line 100: ‘Ultimately, the compound-target and target-pathway networks were constructed’.
19)	Line 101: rephrase as or similar to ‘This study compared the chemical composition and anti-hepatoma activity of D. nobile and D. chrysotoxum extracts, and revealed the potential anti-hepatoma action mechanism of D. chrysotoxum using a multi-target and multi-pathway approach’.
20)	Line 169: positive contrast or positive control?
21)	Line 218: there should be only a ’Results’ section, since there is also a ‘Discussion’ section later on (line 322).
22)	Lines 248 and Lines 252: Dendrobium should be in italics.
23)	Line 278: ‘…showed the strongest anti-hepatoma activity, while the stems extract…’
24)	Line 284: ‘…greater anti-hepatoma activity than that observed in D. nobile, the former species was selected…’
25)	Line 290: ‘The common target may be related to the anti-hepatoma activity of D. chrysotoxum.’ Please, change and amend this sentence; the message is not clear.
26)	Line 291: please provide more details to the readers on what are the nodes/targets (e.g. proteins, biological processes, etc.); please make an effort to deliver a clear message.
27)	‘the topological attribute analysis’? Topological? Please, rephrase or clarify.
28)	Line 372 (Discussion): ‘The research of Yang et al. and Wu et al. indicated that FOXO1 gene was low expressed in liver cancer, which is expected to become a target for diagnosis and treatment of liver cancer’. Please, cite the reference of Yang et al. and Wu et al. here in the sentence. The gene name should be in italics.
29)	‘The influence of D. chrysotoxum to liver cancer may depend on making an impact on EGFR tyrosine kinase inhibitor resistance pathway and FoX O signaling pathway.’ Making an impact? Please, rephrase to be clearer.
30)	Line 379: ‘Meanwhile’ is not correct here. Please amend.
31)	Dendrobium, not Dendrobiums; please control all over the text.
32)	Line 391: ‘Meanwhile, the leaves on the Dendrobiums stem are sometimes peeled for aesthetic reasons’ is somehow redundant with sentence in line 377; please remove or amend.
33)	Line 392: ‘As we can see,’, rephrase as ‘In our study we found that…’
34)	Line 394: ‘Therefore, Dendrobium leaves show potential for the research and development of pharmacological biomolecules’ or similar.
35)	Line 399: the sentence ‘We screened 18 differential ions ([M+H]+ and [M-H]-) in the positive-ion and negative-ion model of UPLC-Q-TOF-MS/MS using OPLS-DA and PCA.’ appears to be too detailed; please consider to rephrase or include it in results. Here you mention ‘18 differential ions ([M+H]+ and [M-H]-)’ for the first time (!), out of any context…please remove and change it.
36)	Line 403: rephrase as or similar to ‘Network pharmacology analysis revealed chrysotobibenzyl, chrysotoxin, moscatilin, gigantol and chrysotoxene as relevant compounds for D. chrysotoxum anti-hepatoma activity’. Is this what the authors mean?
37)	Line 40-7: rephrase as ‘and provided chemical markers as potential candidates for quality control of D. nobile and D. chrysotoxum.’; what do the authors mean by ‘quality control’? Please, be more specific and clearer.
38)	IMPORTANT NOTE: Regarding the authors’ response to one of the reviewers’ question and ‘their use of Lo2 cells (normal human hepatocytes) as normal cells to perform toxicity tests with different concentration gradients of the samples’. Please consider that: (A) this control experiment should be discussed in the text or the data be shown or the previous studies were data can be found should be cited, since the point made by the reviewer is relevant; and (B) if data are shown in the present study as a control, the authors must clarify what types of human samples were used and provide the adequate details on Ethics Approval and Materials and Methods.
```
